# Genomic and phenotypic characterization of multidrug-resistant *Staphylococcus haemolyticus* isolated from burn patients in Chongqing, southwestern China

**DOI:** 10.1128/spectrum.02577-24

**Published:** 2025-04-17

**Authors:** Yuhua Yang, Yali Gong, Naan Zhang, Huagang Peng, Weilong Shang, Yi Yang, Yifan Rao, Zhen Hu, Li Tan, Yuting Wang, Lu Liu, He Liu, Xiaonan Huang, Yulin Zhang, Qiwen Hu, Zhiqiang Yuan, Xiancai Rao

**Affiliations:** 1 Department of Microbiology, College of Basic Medical Sciences, Key Laboratory of Microbial Engineering under the Educational Committee in Chongqing, Army Medical University 12525, Chongqing, China; 2 Institute of Burn Research, State Key Laboratory of Trauma and Chemical Poisoning, Southwest Hospital, Army Medical University 12525, Chongqing, China; 3 Department of Emergency Medicine, Xinqiao Hospital, Army Medical University 12525, Chongqing, China; University of Calgary, Calgary, Alberta, Canada

**Keywords:** *Staphylococcus haemolyticus*, burn unit, whole genome sequencing, ST152, antimicrobial resistance, virulence, hemolytic activity, biofilm formation

## Abstract

*Staphylococcus hemolyticus* is one of the frequently isolated nosocomial pathogens in immunocompromised patients; however, its genotypic and phenotypic characteristics in burn patients remain largely unknown. In this study, 146 *S*. *haemolyticus* strains were collected from the burn unit of a tertiary hospital in Chongqing, southwestern China. Whole genome sequencing was applied to determine the molecular characteristics of these *S. haemolyticus* isolates. Results showed that sequence type 1 (ST1; 26.7%, 39/146) was the most prevalent lineage, followed by clones ST42 (17.8%, 25/146), ST3 (15.8%, 23/146), ST25 (9.6%, 14/146), ST29 (4.8%, 7/146), and newly emerged ST152 (6.2%, 9/146). In terms of phenotypes, 90.4% (132/146) of the *S. haemolyticus* isolates were multidrug-resistant (MDR), and more than 90% strains exhibited resistance to penicillin, erythromycin, oxacillin, and levofloxacin. A total of 145 out of 146 *S*. *haemolyticus* isolates harbored at least 10 virulence factor genes. The tested strains with ST1, ST3, ST25, and ST42 presented similar hemolytic activities and biofilm formation capabilities. All detected ST152 isolates were MDR, corresponding to the number of their antimicrobial resistance genes being the largest among all tested STs. Moreover, ST152 isolates had increased hemolytic capacity and biofilm formation capability compared with their counterpart ST29 strains, the parent lineage of ST152 with a single locus variation. Overall, this study showed the genomic and phenotypic characteristics of 146 *S*. *haemolyticus* strains isolated in Chongqing and found the newly emerged ST152 MDR lineage with hypervirulence, which may call attention to the control of infections caused by MDR *S. haemolyticus* strains in hospitals.

**IMPORTANCE:**

*Staphylococcus haemolyticus* is a common opportunistic pathogen with multidrug resistance in clinical infections. In this study, we analyzed the molecular epidemiological characteristics of 146 *S*. *haemolyticus* strains isolated from the burn patients of a tertiary hospital in Chongqing between 2017 and 2023. The results demonstrated that the phylogenetic evolution of *S. haemolyticus* strains was diverse and plastic. The majority of the isolates were multidrug-resistant (MDR). The virulence genes, resistance elements, and phenotypes, such as hemolysis and biofilm formation, were determined. A new lineage (ST152) that harbored the highest number of resistance genes was characterized to be 100% (9/9) MDR. The prevalence of ST152 *S. haemolyticus* strains suggests a new health threat in terms of control and treatment of coagulase-negative staphylococcal infections in Chongqing. This study provides information for clinical control of *S. haemolyticus* dissemination and infection in hospitals.

## INTRODUCTION

Coagulase-negative staphylococci (CoNS) are nonpathogenic commensal bacteria that colonize the surface of human skins and mucous membranes (1, 2). However, CoNS, such as *Staphylococcus epidermidis* and *Staphylococcus haemolyticus*, have become the most frequently isolated microbes in the immunocompromised population, including neonates, the elderly, and patients with cancer (3, 4). Epidemiological studies have revealed that *S. haemolyticus* is the second most commonly isolated pathogen among clinical CoNS infections (5), causing a variety of diseases, including conjunctivitis, sepsis, otitis, diabetic foot ulcer, and urinary tract infections (6–10). Furthermore, clinical outbreaks of *S. haemolyticus* infection have been documented in patients with extensive burns (11, 12). In addition, *S. haemolyticus* can infect animals and plants and survive in various environments (13–15). However, the molecular characteristics of *S. haemolyticus* isolates are seldom investigated.

The prevalence of multidrug-resistant (MDR) *S. haemolyticus* limits the choice of antimicrobial agents in clinical settings (16, 17). *S. haemolyticus* can act as a genetic reservoir for antimicrobial-resistance (AMR) and virulence factor genes, which are transmitted through diverse mobile genetic elements to other bacteria (18, 19). Moreover, *S. haemolyticus* can obtain genetic elements from other bacteria through horizontal gene transfer (20–22), and this frequent genetic exchange contributes to the genomic plasticity and genome rearrangement of *S. haemolyticus* (23–25). Therefore, the genomic evolution of *S. haemolyticus* is dynamic, and its clonal lineages dominating in certain areas are also changeable. Several studies have demonstrated the emergence of methicillin-resistant *S. haemolyticus* isolates in neonatal intensive care units, including those of the late-onset sepsis-associated ST2, ST4, and ST29 clonal lineages (2, 8, 26). Qin et al. (17) conducted a genomic analysis of 97 *S*. *haemolyticus* isolates and characterized ST42 as a MDR and pathogenic lineage disseminated among patients and hospital environments in Guangzhou, southeastern China. However, data on the genome and phenotypes of *S. haemolyticus* clinical strains are limited.

This retrospective study aims to characterize the genomic and phenotypic characteristics of *S. haemolyticus* isolates from burn patients. A total of 146 *S*. *haemolyticus* isolates collected between 2017 and 2023 in Chongqing, southwestern China were conducted. Whole genome sequencing (WGS) was performed to reveal the genomic features of the *S. haemolyticus* isolates. The virulence genes, resistance genes, as well as bacterial phenotypes like hemolysis and biofilm formation of *S. haemolyticus* isolates, were also determined. The obtained data will facilitate the control and treatment of *S. haemolyticus* infections in hospitals.

## RESULTS

### Clinical characteristics of the *S. haemolyticus* isolates

**Fig 1:**
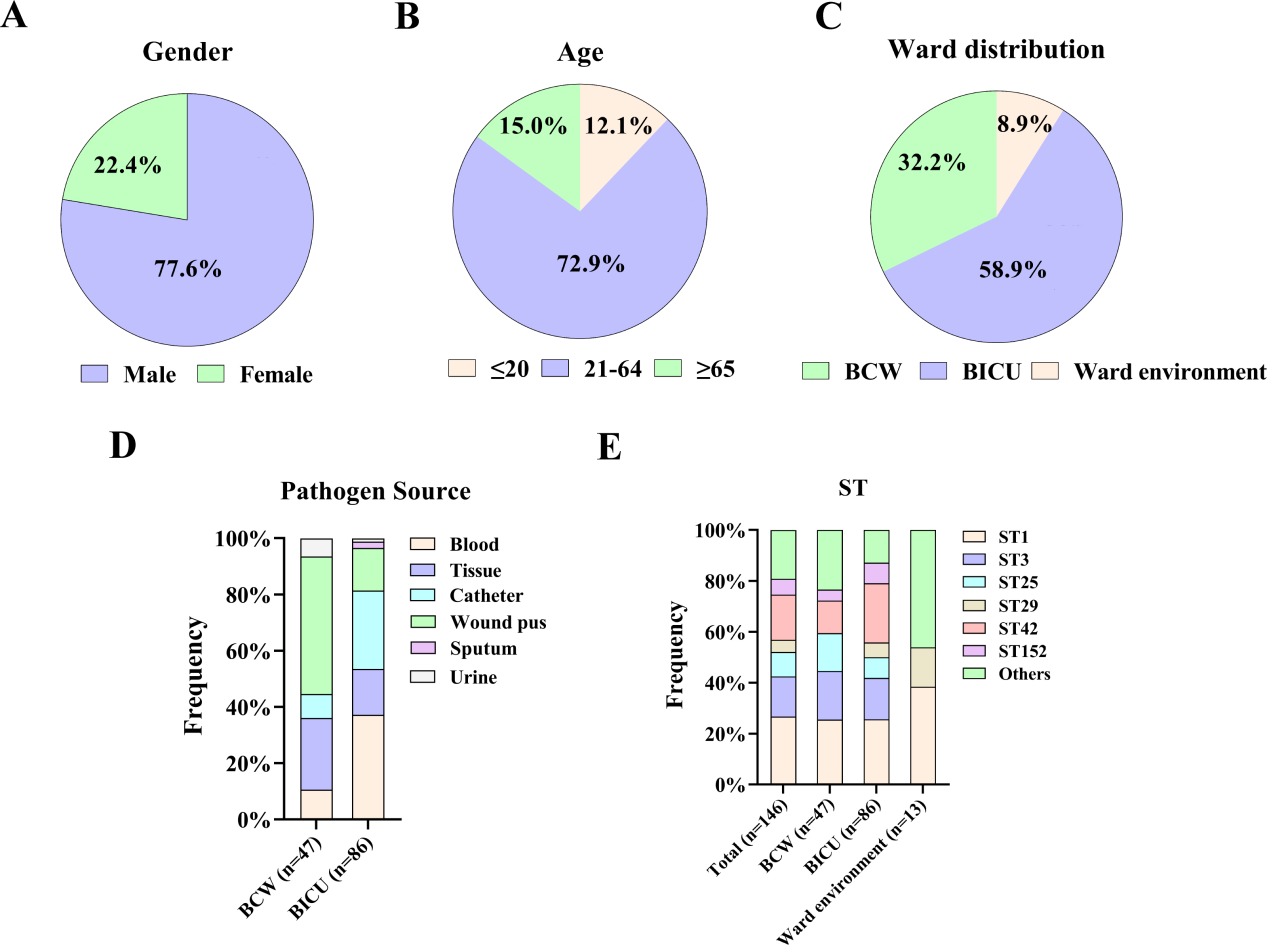
Clinical information of burn inpatients, strain sources, and ST distribution of the *S. haemolyticus* strains. Clinical information of *S. haemolyticus*-infected inpatients classified by (**A**) gender and (**B**) age. (**C**) *S. haemolyticus* strain distribution in ward. (**D**) Distribution of specimen sources in the *S. haemolyticus* isolates. (**E**) Distribution of STs in the *S. haemolyticus* strains.

Samples were collected from multiple sites of the inpatient body and hospital environments to clarify the distribution of *S. haemolyticus* in the hospital burn unit. A total of 146 *S*. *haemolyticus* strains were isolated from 107 burn inpatients and ward environments between 2017 and 2023. Male patients were more susceptible to *S. haemolyticus* infections than females (77.6% versus 22.4%) (Fig. 1A). With regard to age, most *S. haemolyticus* strains (72.9%, 78/107) were isolated from inpatients aged between 21 and 64 years (Fig. 1B). In particular, 47 strains were obtained from the patients hospitalized in burn common ward (BCW; 32.2%, 47/146), 86 strains from the burn intensive care unit (BICU; 58.9%, 86/146), and 13 isolates from ward environments (Fig. 1C). Furthermore, *S. haemolyticus* strains in BCW were mainly isolated from wound pus (48.9%, 23/47), followed by tissue (25.5%, 12/47), blood (10.6%, 5/47), catheter (8.5%, 7/47), and urine (6.4%, 3/47). In BICU, 37.2% (32/86) isolates were derived from blood, 27.9% (24/86) from catheter, 16.3% (14/86) from tissue, 15.1% (13/86) from wound pus, and 3.4% (3/86) from sputum and urine (Fig. 1D). The environmental sampling sites included bedside, doorknob, infusion pump, and electrocardiographic monitor, and desirable strains were obtained. These findings revealed that *S. haemolyticus* was widely distributed throughout the hospital units.

### Genomic diversity of the *S. haemolyticus* isolates

WGS was performed to characterize the molecular epidemiology of *S. haemolyticus* isolates. Fifteen known STs were identified in 123 out of 146 sequenced isolates (84.2%). The top five STs, namely, ST1 (26.7%, 39/146), ST42 (17.8%, 25/146), ST3 (15.8%, 23/146), ST25 (9.6%, 14/146), and ST29 (4.8%, 7/146), accounted for 74.0% (108/146) of the strains tested (Fig. 1E; Table S1). ST1 (25.5%) and ST3 (19.1%) were the dominant clones in BCW, and ST1 (25.6%) and ST42 (23.3%) were the main clones in BICU. Meanwhile, ST1 (38.5%) and ST29 (15.4%) were the main environmental strains. The 23 remaining unidentified strains were analyzed by uploading their alleles, and nine strains (6.2%, 9/146) were assigned as a new type labeled ST152, which is a single-locus variant of the ST29 clone (ribose_ABC-9 in ST152 versus ribose_ABC-4 in ST29) (Table S2). ST152 was classified into clonal complex 3 (CC3), which contains the main epidemic clonal lineages, such as ST1, ST3, ST25, ST29, and ST42.

**Fig 2:**
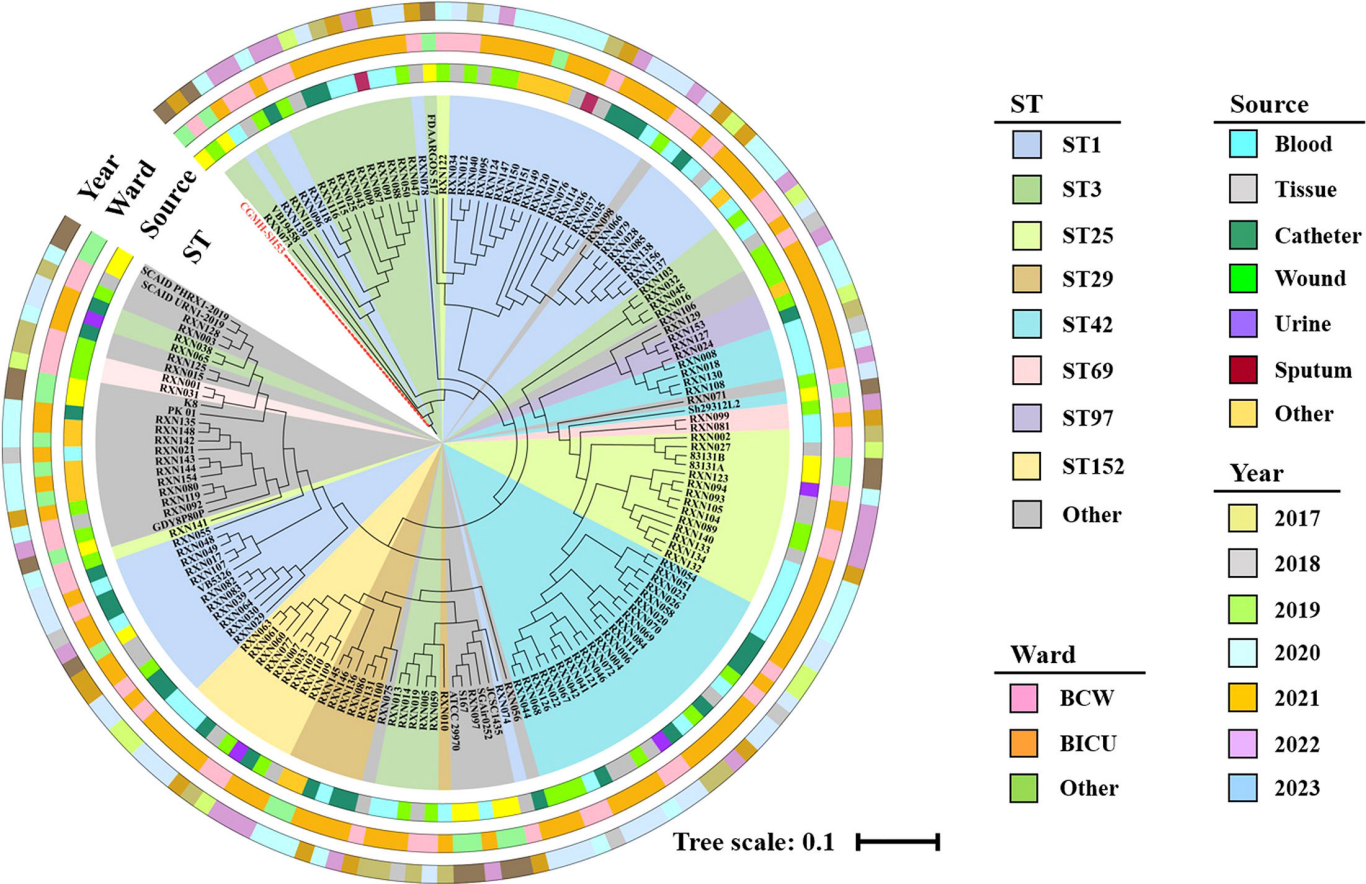
Approximate maximum-likelihood phylogenetic tree of *S. haemolyticus* strains isolated from the burn unit based on core-genomic SNPs. The genome sequence of CGMH-SH53 (a CC3-ST3 strain) was used as the reference. The tree was generated using the iTOL online platform (https://itol.embl.de/). The total branches of the six clades are shown in diverse colors. The inner circle indicates different STs, and the sample source, ward distribution, and the time of strain collected were colored and mapped in the outer rings.

A phylogenetic tree based on 16,221 core genomic single nucleotide polymorphisms (SNPs) was generated using the maximum likelihood (ML) method. Figure 2 shows a long-branching and scattered population structure classifying the distinct phylogenetic lineages. A scattered phylogeny was observed for the prevalent STs, such as ST1, ST3, ST25, ST29, and ST42, indicating a high level of genetic diversity within the *S. haemolyticus* isolates. The number of SNPs within the same branch was relatively similar, and the strain distribution was well aligned with the ML tree. The SNP calling analysis revealed that strains of the same ST with varied SNPs were clustered in one branch, except for ST1, ST3, and ST42 (Fig. S1 to S6). The ST1 strains were primarily grouped into three distinct branches. A similar trend was noted for the ST3 and ST42 strains (Fig. 2). These data suggested a high degree of genomic plasticity within the tested *S. haemolyticus* clones. Seven ST29 strains were clustered in the same branch, with the exception of strain RXN010 (Fig. 2), and less than 35 SNPs differed among these ST29 strains (Fig. S4). Four ST1 strains derived from a patient (strain RXN124 from wound pus) and his hospitalization environments (RXN147, RXN149, and RXN150 isolated from the rail, head, and foot of the patient bed, respectively) were clustered in a single branch with SNP numbers of less than 3 (Fig. S1). Importantly, one ST1 environmental strain RXN151 from another ward was also clustered in the same branch, indicating a possible transmission of *S. haemolyticus* from patients to the environments. By contrast, three strains from the same patient belonged to various STs (RXN100, ST29 from catheter; RXN107, ST1 from blood; and RXN111, ST42 from wound pus), indicating the cross-infection of a single patient within 4 months of hospitalization.

A pan-genome analysis of the 146 *S*. *haemolyticus* isolates and 16 reference strains (Table S3) revealed a hierarchical genomic architecture comprising four distinct categories (Fig. S7A), namely, 1,274 core genes (presenting in 99–100% of strains), 462 soft core genes (95–99% prevalence), 1,078 shell genes (15–95% occurrence), and 7,059 cloud genes (less than 15% distribution). A gene accumulation curve analysis revealed a stable distribution of the conserved 1,736 core genes and an increasing number of the open pan-genomic genes with the addition of bacterial strains (Fig. S7B). The distribution pattern of all 9,873 pan-genomic genes for each *S. haemolyticus* strain is shown in Fig. S7C. The conserved core genes were clustered. These data demonstrated that the core and soft-core genes may contribute to determining *S. haemolyticus* species, while the shell genes may correlate with STs, and cloud genes may be the key determinants for strain identification within the same ST.

### Antimicrobial susceptibility and AMR gene carriage in the *S. haemolyticus* isolates

**Fig 3:**
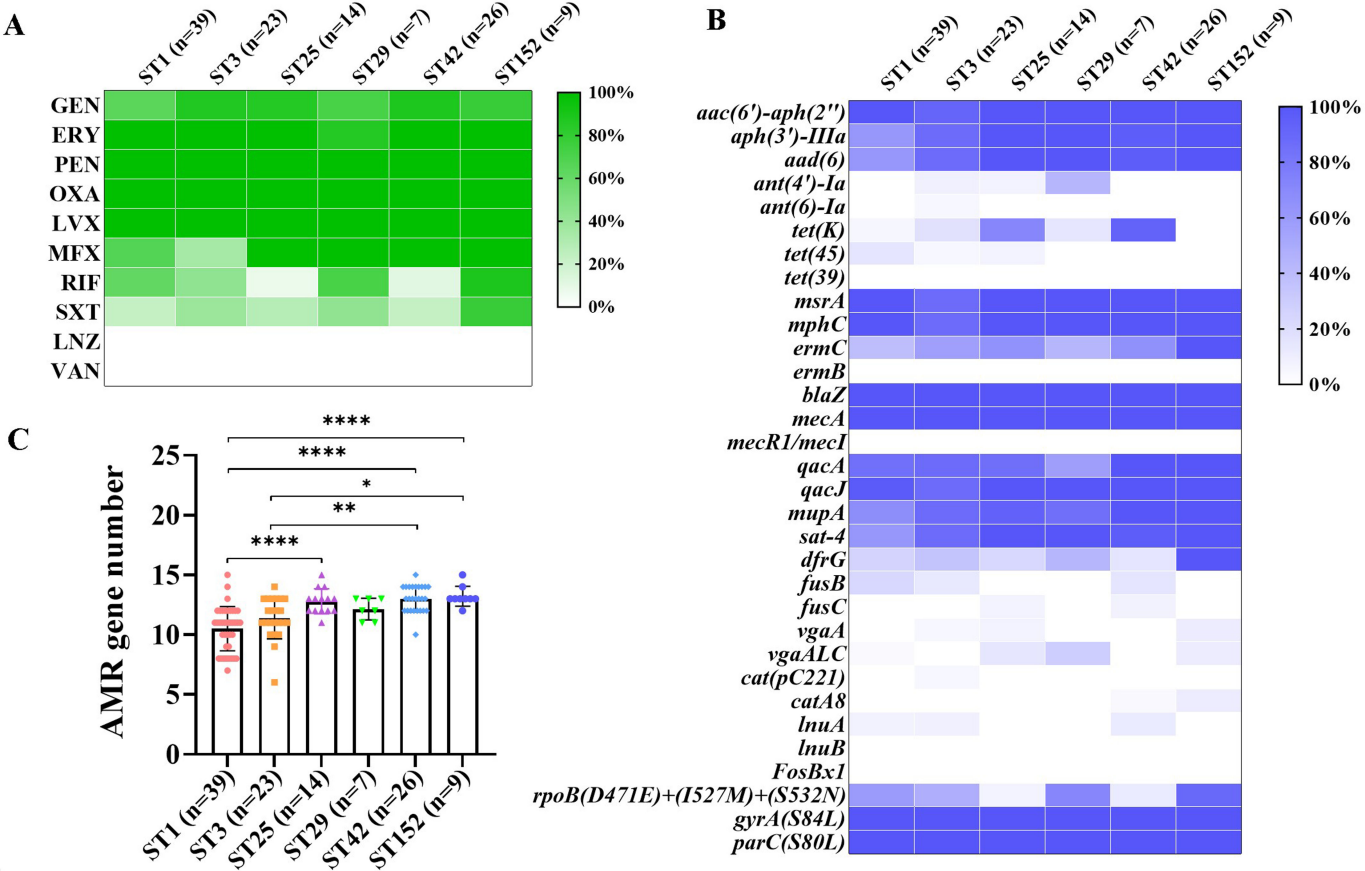
Antimicrobial resistance and AMR gene carriage of the *S. haemolyticus* isolates belonging to major STs. (**A**) Antimicrobial resistant rates in *S. haemolyticus* isolates of the certain STs. (**B**) Distribution of AMR genes in *S. haemolyticus* strains of the major STs. (**C**) Number of AMR genes carried by *S. haemolyticus* of the major STs. The data are shown as mean ± standard deviation. Statistical analysis was performed using one-way analysis of variance. ****P < 0.0001, ***P < 0.001, **P < 0.01, and *P < 0.05. GEN, gentamicin; ERY, erythromycin; PEN, penicillin; OXA, oxacillin; LVX, levofloxacin; MFX, moxifloxacin; RIF, rifampicin; SXT, trimethoprim/sulfamethoxazole; LZD, linezolid; and VAN, vancomycin.

Antimicrobial susceptibility determination revealed that 90.4% (132/146) of the *S. haemolyticus* isolates were MDR, and all the strains were susceptible to linezolid and vancomycin (Fig. 3A). Most of the *S. haemolyticus* strains exhibited high resistance to penicillin (98.6%, 144/146), erythromycin (97.9%, 143/146), oxacillin (95.9%, 140/146), levofloxacin (91.1%, 133/146), gentamicin (69.9%, 102/146), and moxifloxacin (67.8%, 99/146). Meanwhile, the resistance rate to rifampicin and trimethoprim/sulfamethoxazole was less than 40% (Table S4). The *S. haemolyticus* strains with various STs exhibited comparable resistance rates to diverse antibiotics, except for ST25 and ST42 isolates that had low resistance to rifampicin (<15%). Similar to those from the ward environments, strains derived from BCW and BICU presented resistance phenotypes to drugs, except gentamicin and moxifloxacin.

**Fig 4:**
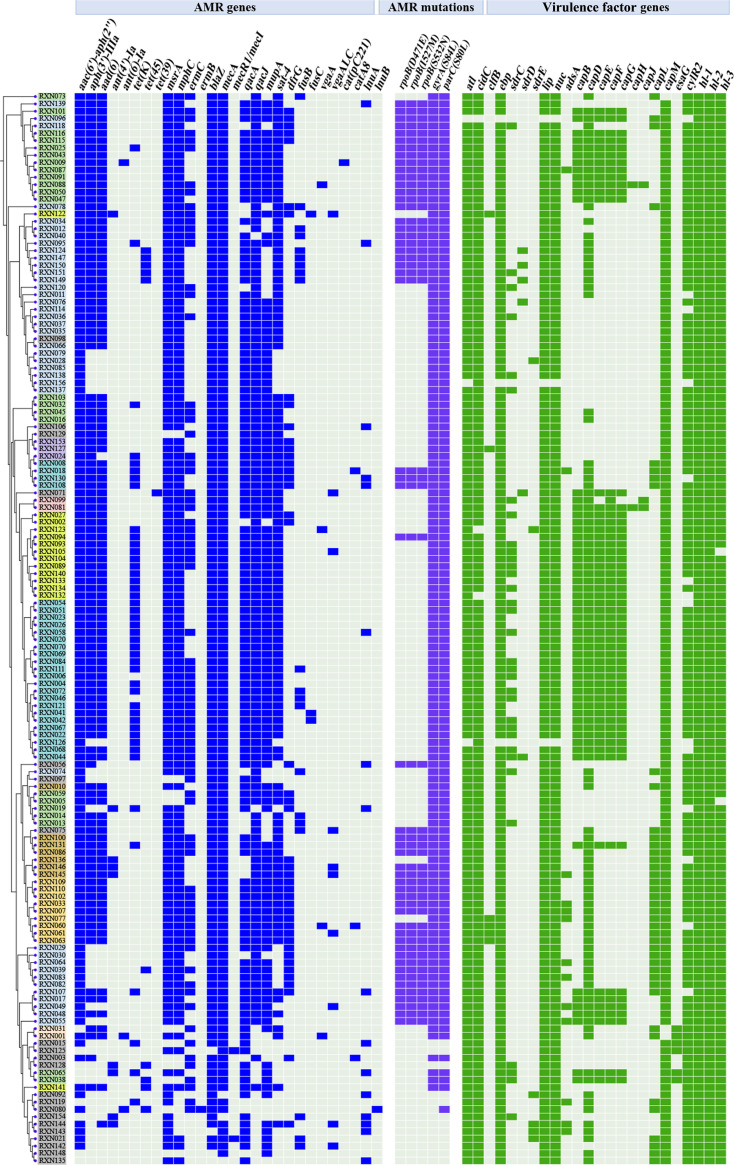
AMR gene carriage, AMR genetic point mutation, and virulence factor gene distribution of the 146 *S*. *haemolyticus* isolates. The presence of an AMR gene, a point mutation, and a virulence factor gene in a strain is shown in blue, purple, and green, respectively, while the absence of an AMR gene, point mutation, and virulence factor gene in the strain is indicated in gray.

The presence of AMR genes in 146 *S. haemolyticus* strains was detected using WGS data. Thirty-two AMR genes were predicted among the strains of interest (Fig. 3B), and the new ST152 strains had the highest number of AMR genes (Fig. 3C). More than 80% of the *S. haemolyticus* strains carried resistance genes *mecA*, *blaZ*, *aac(6′)-aph(2*″), *msrA*, *mphC*, *qacA*, *qacJ*, and *mupA* (Fig. 4). Moreover, 37.0% (54/146) of the *S. haemolyticus* strains had three mutations in the *rpoB* gene, namely, D471E, I527M, and S532N, which are responsible for rifampicin resistance. The majority of rifampicin-resistant strains (77.8%, 42/54) also carried the *rpoB*(S796A) mutation. Most strains (91.1%, 133/146) exhibited fluoroquinolone resistance caused by *gyrA*(S84L) and *parC*(S80L) mutations.

All the ST152 strains were MDR and carried 16.1 ± 0.9 resistance genes, including *aac(6′)-aph(2*″), *aph(3′)-IIIa*, and *aad (6*) for aminoglycosides, *msrA*, *mphC*, and *ermC* for macrolides, *blaZ* and *mecA* for beta-lactam, *qacA* and *qacJ* for quaternary ammonium compounds, *mupA* for mupirocin, *sat-4* for nucleoside antibiotic, and *dfrG* for trimethoprim. Point mutations were also found in ST152 isolates, such as *rpoB*(D471E/I527M/S532N) in 88.9% (8/9) of the strains, *gyrA* (S84L; 100%, 9/9), and *parC* (S80L; 100%, 9/9). These data confirmed the resistance phenotype of the ST152 isolates *in vitro* (Fig. 3A), suggesting that ST152 is an emerging high-risk lineage.

### Virulence factor gene profiles of the *S. haemolyticus* isolates

Twenty-three virulence factor genes associated with *S. haemolyticus* pathogenesis were found (Fig. 4). Among these genes, *atl* (autolysin; 97.9%, 143/146), *cidC* (adherence; 97.9%, 143/146), *ebp* (cell surface elastin binding protein; 97.9%, 143/146), *lip* (lipase; 97.9%, 143/146), *nuc* (nuclease; 97.9%, 143/146), *capM* (capsule synthesis; 97.9%, 143/146), and *cylR*2 (cytolysin; 90.4%, 132/146) were highly prevalent. Evidence of the presence of virulence factor genes in the BCW and BICU strains was not found. In addition, the strains isolated from the ward environments lacked the genes *capB*, *capE*, *capF*, *capG*, *clfB*, and *esaG*. In *S. haemolyticus* CGMH-SH53, two hemolysin family proteins (WP_01693051.1 and WP_011276453.1) and one hemolysin III family protein (WP_011275183.1) were annotated (27), and we designated these genes as *hl-1*, *hl-2*, and *hl-3*, respectively. Sequence alignment was performed across all 146 *S*. *haemolyticus* strains, and the results revealed that all tested strains harbored *hl-1* and *hl-2* genes, while 98.6% (144/146) of the tested isolates carried *hl-3*, except for strains RXN005 and RXN105.

**Fig 5:**
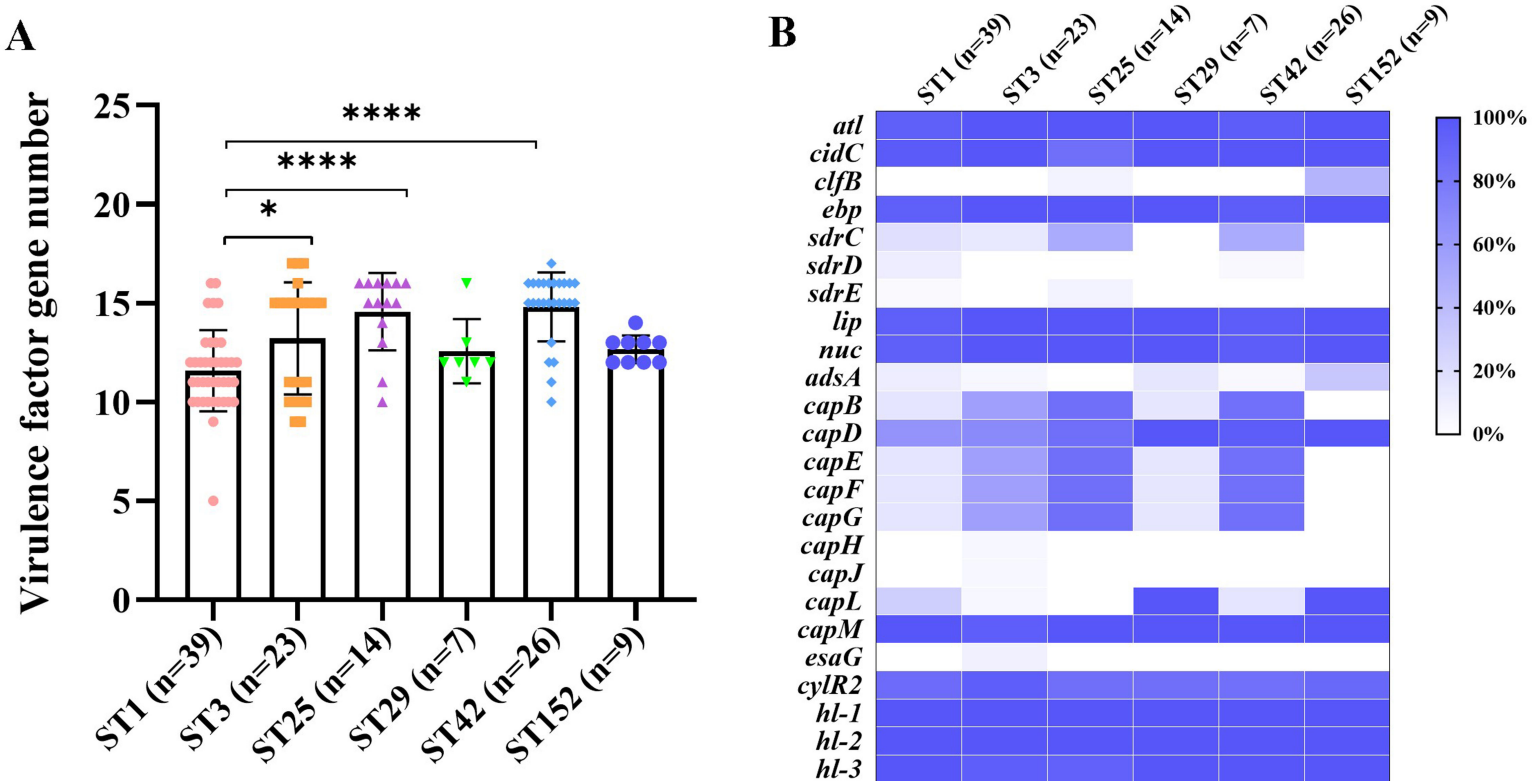
Distribution of the virulence factor genes among *S. haemolyticus* isolates of the major STs. (**A**) Number of virulence factor genes carried by *S. haemolyticus* of the major STs. The data are shown as mean ± standard deviation. Statistical analysis was performed using one-way analysis of variance. ****P < 0.0001 and *P < 0.05. (**B**) Distribution of virulence factor genes in the major STs of *S. haemolyticus* strains. The percentage of a virulence factor gene in certain ST was indicated.

The presence of virulence factor genes among the *S. haemolyticus* strains of the major STs is indicated in Fig. 5. The ST3, ST25, and ST42 strains carried more virulence factor genes than the ST1 strains (Fig. 5A). The adhesion-related genes *sdrC*, *sdrD*, and *sdrE* were completely absent in the ST29 and ST152 strains (Fig. 5B). All (9/9) the ST152 strains harbored *capD*, *capL*, and *capM* genes, and most of them carried the *clfB* gene (66.7%, 4/6). The diversity and function of virulence factor genes in *S. haemolyticus* isolates need further investigation.

### Hemolytic activity and biofilm formation capacity of the *S. haemolyticus* strains

**Fig 6:**
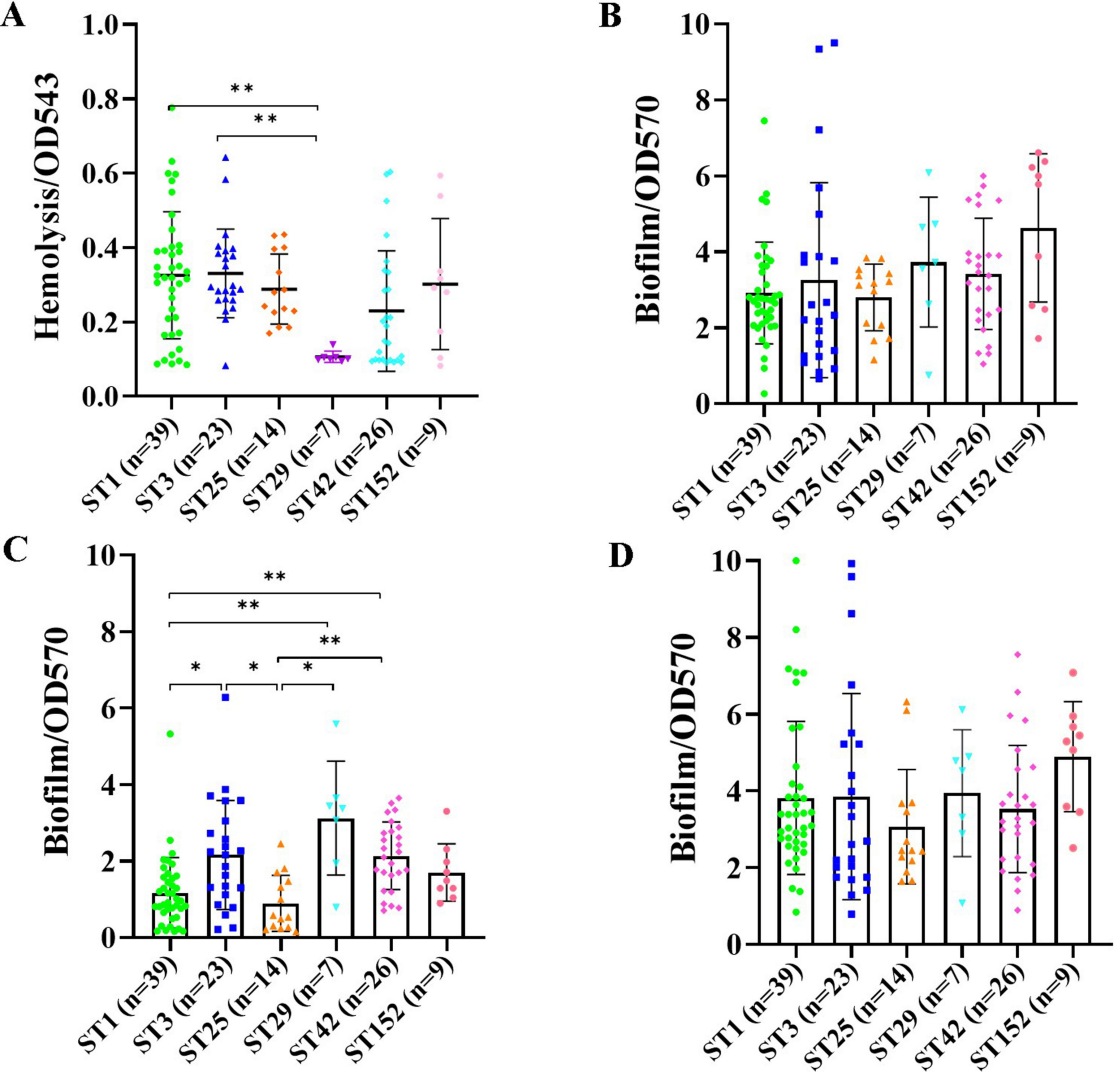
Hemolytic activity and biofilm formation capacity of the *S. haemolyticus* isolates belonging to major STs. (**A**) Erythrocyte lysing ability of the major ST strains. The hemolytic activity was determined by a test tube method at an absorbance of 543 nm. Detection of biofilm formation ability of *S. haemolyticus* strains of the major STs cultured in (**B**) BHI, (**C**) BHI_NaCl_, and (**D**) BHI_glu_. Biofilm formation capacity was detected by semiquantitative crystal violet method at an absorbance of 570 nm. Each experiment was independently repeated three times. The data are shown as mean ± standard deviation. Statistical analysis was performed using one-way analysis of variance. **P < 0.01 and *P < 0.05.

The hemolytic activity of each strain was examined using rabbit erythrocytes. The results showed that the *S. haemolyticus* isolates of most STs displayed comparable hemolytic capacities (Fig. 6A). However, the hemolytic activity varied among the ST1, ST42, and ST152 isolates. The ST1 and ST3 strains showed a remarkably stronger hemolytic activity than the ST29 isolates (*P* < 0.01). As a variant of ST29, the ST152 strains presented increased hemolysis capacity compared with the ST29 isolates [optical density value at 543 nm (OD_543_) 0.30  ±  0.18 versus 0.11  ±  0.01], although no significant difference was observed.

Glucose and NaCl affect bacterial biofilm formation (17, 28). Thus, the *S. haemolyticus* strains were each cultured with BHI, BHI with 1% glucose (BHI_glu_), and 3% NaCl (BHI_NaCl_). Semiquantitative crystal violet assay was then conducted to test the biofilm formation capacity of these bacteria under various conditions. Compared with the *S. haemolyticus* strains cultured in BHI (Fig. 6B), those grown in BHI_NaCl_ showed reduced biofilm formation capabilities (Fig. 6C), and those cultivated in BHI_glu_ exhibited enhanced biofilm formation, especially the ST1 strains (Fig. 6D). The biofilm formation capacities of 24 *S*. *haemolyticus* strains were suppressed when cultured with BHI_NaCl_ but were restored when normal BHI or BHI_glu_ medium was used. The ST3, ST29, and ST42 isolates produced greater biofilm than ST1 and ST25 strains when cultured in BHI_NaCl_ (*P* < 0.05). Furthermore, the ST152 strains grown in BHI and BHI_glu_ showed a slightly higher biofilm formation capability than their counterpart ST29 isolates. Overall, these data indicated that *S. haemolyticus* strains of the major STs exhibited varied hemolytic activity and biofilm formation capability, which is consistent with their virulence factor gene profiles.

## DISCUSSION

As an important nosocomial infection microorganism, *S. haemolyticus* can cause outbreaks in different hospital departments, such as burn and neonatal units (2, 12). Most victims suffering from *S. haemolyticus* infections are immunocompromised, such as neonates, burn patients, and individuals with cancer. Burn patients may have a compromised skin barrier after debridement, skin transplantation, and dressing change. These burn-related operations may increase the risk of nosocomial infections (29). However, the genomic and phenotypic characteristics of *S. haemolyticus* clinical isolates remain largely unclear. In this study, the molecular characteristics of 146 *S*. *haemolyticus* strains collected from a hospital burn unit between 2017 and 2023 were analyzed. Results demonstrated that different *S. haemolyticus* genotypes are strongly associated with clinical infections. *S. haemolyticus* can be detected in various samples (blood, urine, catheter, tissue, and wound pus) from burn inpatients and their hospitalization environments, indicating a possible cross transmission among patients, nursing staff, and the ward environments. Noshak et al. (30) reported that the transmission of CoNS pathogens among healthcare workers, shared equipment, and patients can occur in the ICUs. We found that *S. haemolyticus* strains with different genotypes (strains RXN008, RXN009, and RXN010) or diverse drug-resistant phenotypes (strains RXN093 and RXN094) can be isolated from a single patient, reflecting the high complexity of *S. haemolyticus* transmission in hospitals. We characterized ST1 as the most prevalent clone, and this result was contradictory to a previous report that showed ST42 was the dominant clone in the burn unit of a hospital in Taiwan, China (12). The mechanism underlying this variation of the most popular clones is unclear. The limited strains used in the two studies (146 versus 97) may lead to a bias, and more strains are needed to figure out the most popular *S. haemolyticus* clones in certain regions. Moreover, the geographical and cultural differences could be vital factors to select the most prevalent bacterial lineages in hospitals (17).

According to the ML tree generated using core-genomic SNPs, *S. haemolyticus* isolates belonging to the same ST could distribute in diverse clusters, such as ST1, ST3, and ST42, suggesting the divergence or recombination of housekeeping genes in certain STs (31, 32). The strains with the same ST, such as ST1, could be isolated from a single patient and his ward environments. Moreover, an individual burn patient could be infected with *S. haemolyticus* isolates of diverse STs during hospitalization. These data showed a cross-transmission of *S. haemolyticus* in the investigated burn units. The pan-genome analysis revealed that the shell genes may correlate with bacterial STs, while the cloud genes could contribute to the intra-ST distribution of *S. haemolyticus* isolates. The highly plastic nature of the cloud genes suggested substantial horizontal gene transfer events and genomic rearrangements. The variation of genes contributes to the evolution of bacterial lineages (33, 34), and recombination plays a decisive role in bacterial resistance, adaptation, and colonization (35). However, the evolutionary and ecological significance of the cloud genes needs further investigation.

*S. haemolyticus* has the highest antibiotic resistance among CoNS in the hospital environment, and its notorious AMR gene reservoirs can transmit resistance genes to other bacterial species (7, 33). Our data showed that 90.4% (132/146) of the *S. haemolyticus* isolates were MDR, which is similar to the previous studies (2, 8). Most mutations in the *rpoB* gene are responsible for rifampicin resistance in bacteria (36, 37), and those in topoisomerase IV or DNA gyrase result in resistance to quinolone antibiotics (38, 39). Our data revealed that the new ST152 *S. haemolyticus* strains carried the largest number of AMR genes and exhibited the highest rate of resistance to rifampicin and trimethoprim/sulfamethoxazole among all the STs. Moreover, 100% of the ST152 isolates (9/9) harbored S84L and S80L mutations in their *gyrA* and *parC* genes, respectively, which is consistent with the previous studies (39, 40), suggesting that ST152 *S. haemolyticus* with MDR could be an important risk factor for the outbreaks in hospitals.

The virulence of *S. haemolyticus* isolates is seldom studied (28). Rabbit erythrocyte lysis experiments revealed variation in hemolytic activity among the *S. haemolyticus* strains of the major STs, except for ST29 strains, which showed comparable and low hemolytic capabilities. By contrast, the hemolysis capacity of the ST152 strains increased compared with that of the ST29 isolates. Genomic analysis of virulence determinants revealed that hemolysin-associated genes were ubiquitously present across the *S. haemolyticus* strains. However, phenotypic characterization using a rabbit erythrocyte hemolysis assay demonstrated distinct profiles: the strains of ST29 lineage exhibited negligible hemolytic activity, while the isolates of other predominant STs displayed variable hemolytic potentials. Notably, ST152 strains showed enhanced hemolytic capacity compared to its progenitor ST29. The discrepancy between genotype and phenotype of the tested *S. haemolyticus* strains may be ascribed to the epigenetic regulation or post-transcriptional modification of hemolysin-related gene products under *in vitro* culture conditions. A previous study revealed that *S. haemolyticus* isolates in the absence of α-toxin and PSM peptides exhibited considerable hemolytic activities (41). The mechanisms underlying hemolytic regulation in *S. haemolyticus* are obscure and necessitate a comprehensive characterization of more isolates using integrated genomic and proteomic approaches.

Polysaccharide intercellular adhesin-dependent pathway plays an essential role in the biofilm formation of *S. aureus* and *S. epidermidis*; *S. haemolyticus* biofilms are mainly composed of extracellular DNA and proteins (42–44). The *nuc* and *atl* genes responsible for eDNA release and adherence-related genes, such as *clfB*, *ebp*, and *sdrCDE*, were detected in most of the *S. haemolyticus* strains (Fig. 5). Glucose can promote the biofilm formation of *S. haemolyticus* (28). We found that the biofilm formation of *S. haemolyticus* isolates was remarkably affected by the culture medium. *S. haemolyticus* strains grown in BHI_NaCl_ revealed weakened biofilm formation compared with those cultivated in normal BHI and BHI_glu_ (Fig. 6). In addition, ST152 strains cultured in BHI and BHI_glu_ exhibited a slightly higher biofilm formation capability than the ST29 isolates. The increase in the hemolytic activity and biofilm formation capability of ST152 *S. haemolyticus* isolates relative to those of their counterpart ST29 strains may contribute to the prevalence of ST152 in the hospital.

In conclusion, we provided molecular insights into the *S. haemolyticus* isolates from burn patients and the ward environment. Several major STs, including ST1, ST3, ST25, ST42, and the newly identified ST152, were disseminated in the hospital. All the ST152 strains were MDR and harbored the highest number of AMR genes. ST1 was the main prevalent lineage of *S. haemolyticus* and had the highest hemolytic capacity. The ST152 strains revealed a relatively high biofilm formation capability. The prevalence of the MDR ST152 lineage of *S. haemolyticus* suggests a new health threat to the control and treatment of CoNS infections.

## MATERIALS AND METHODS

### Study design and collection of *S. haemolyticus* isolates

A total of 146 *S*. *haemolyticus* isolates were isolated from inpatients and the ward environment between 1 January 2017 and 30 June 2023 in a hospital burn unit in Chongqing, southwestern China.

The clinical data, including demographic data (gender and age), clinical features (burn etiology and burn area), and sample sources, were obtained from medical records, and the personal information of the patients was anonymized. A total of 133 *S*. *haemolyticus* isolates were taken from inpatients (hospitalized in BCW and BICU), and 13 strains were obtained from the ward environment. Bacteria were cultured in Columbia sheep blood agar plates (5%, v/v; Biomérieux, Inc., USA) at 37°C for 18–24 h under aerobic conditions. Routine methods, such as Gram staining, catalase assay, and coagulase test, were used to further confirm the *S. haemolyticus* isolates. All isolates were kept frozen at −80°C in brain heart infusion (BHI; Oxford, UK) medium supplemented with 40% glycerol.

### Antimicrobial susceptibility testing

Antimicrobial susceptibilities of the *S. haemolyticus* strains were measured with VITEK-2 compact system analysis following the instructions of the manufacturer. The minimum inhibitory concentrations of the selected antimicrobial agents against each *S. haemolyticus* isolate were determined by the broth dilution method following the Clinical and Laboratory Standards Institute document M100. The tested antimicrobial agents included penicillin, oxacillin, gentamicin, levofloxacin, moxifloxacin, erythromycin, linezolid, vancomycin, rifampicin, and trimethoprim/sulfamethoxazole. *Staphylococcus aureus* strain ATCC29213 was used as a control.

### Whole genome sequencing, strain typing, and phylogenetic analysis

All 146 *S*. *haemolyticus* strains were cultivated aerobically at 37°C for 18–24 h on Columbia sheep blood agar plates. A single colony was then picked up and inoculated into fresh BHI medium cultured at 37°C overnight. The culture was centrifuged (5,000 × *g*, 4°C, 10 min), and bacterial cells were washed once with phosphate-buffered saline (PBS, pH 7.2). The genomic DNA of each *S. haemolyticus* strain was extracted using a QIAamp DNA Mini Kit (Qiagen, Germany) following the manufacturer’s instructions. The genome was sequenced on an Illumina NovaSeq platform (Illumina, Inc., USA) using the 2 × 150 bp paired-end mode. The low-quality and adapter sequences were removed to obtain clean reads. The reads were assembled into contigs using CLC Genomics Workbench software (version 23.0; CLC bio) by applying the *de novo* assembly mode.

Multi-locus sequence typing (MLST) that relies on the variation of seven housekeeping alleles (*arcC*, *SH_1200*, *hemH*, *leuB*, *SH1431*, *cfxE*, and *Ribose-ABC*) was performed for each *S. haemolyticus* strain based on its genome. The assignment of STs was carried out utilizing the pubMLST database (https://pubmlst.org/; 45). In addition, any newly identified alleles and profiles were submitted to this database for analysis. The SNP variant calling method and workflow were performed. Recombination-filtered core genome SNPs generated by ParSNP were used to construct the approximate ML phylogenetic tree of the 146 isolates by using FastTree 2 (46, 47). The phylogenomic tree was visualized and modified using iTOL (https://itol.embl.de/) (48). Core- and pan-genome analyses were conducted using Roary pipeline 3.13.0 with default settings, utilizing annotated assemblies in GFF3 format obtained from the Prokka results as previously described (49).

In addition, the AMR genes were searched using the Comprehensive Antibiotic Resistance Database v3.2.6 with Resistance Gene Identifier 6.0.1 (50). Potential virulence factor genes across all genomes were detected by homology searches using the Virulence Factor Database (51). The hemolysin genes in 146 *S*. *haemolyticus* isolates were analyzed using the reference sequences of strain CGMH-SH53*,* including those encoding hemolysin family proteins (WP_01693051.1 and WP_011276453.1) and hemolysin III family protein (WP_011275183.1). We designated these hemolysin genes as *hl-1*, *hl-2*, and *hl-3*, respectively.

### Hemolytic experiment

The erythrocyte lysis test was performed as previously described (52). Briefly, *S. haemolyticus* strains were cultured in BHI at 37°C for 16 h. Then, the culture was inoculated (1:100 dilution) into a fresh BHI medium and cultured overnight. Afterward, the culture was centrifuged at 12,000 × *g* at 4°C for 5 min, and 1 mL of the culture supernatant was collected. The defibrinated rabbit erythrocyte suspension (6%, v/v) was prepared with PBS, and 100 µL of erythrocytes was added to 100 µL of the bacterium–culture supernatant, and then incubated at 37°C for 30 min. Red blood cells treated with culture supernatants of the *S. aureus* Newman (provided by Prof. Lu Yu, Jilin University, China) and double-distilled water (dd_H2O_) were used as positive controls, while those treated with PBS were used as negative controls. The OD_543_ values were read by a micro-enzyme-linked immunosorbent assay reader (Thermo Scientific, USA). Three replicates were made for each experiment, and the test was repeated three times.

### Biofilm formation assay

The semiquantitative crystal violet assay for the *S. aureus* biofilm was performed as previously reported (28, 42, 53). Briefly, *S. haemolyticus* strains were cultured in BHI at 37°C for 16 h. Then, bacterial cells were adjusted with BHI broth to an OD_600_ of 0.5, and 20 µL bacterial solution was added to the 96 flat-bottom microtiter plates prefilled with 180 µL of BHI, BHI_glu_ [BHI with 1% (m/v) glucose], and BHI_NaCl_ [BHI added with 3% (m/v) NaCl]. After incubation at 37°C for 24 h, the culture supernatants were removed. The plate wells were washed three times with PBS, dried, and fixed with 2.5% (v/v) glutaric dialdehyde (Chongqing Boer Biotech Co., China) for 90 min. After washing twice with PBS, 200 µL of 0.1% (m/v) crystal violet was added to each well and stained for 15 min at room temperature. Finally, the plates were washed with slow water, and 200 µL 33% (v/v) glacial acetic acid was added to dissolve crystal violet. The OD_570_ values were recorded using a microplate reader. *S. epidermidis* strain ATCC 35984 (provided by Prof. Wenchang Yuan, Guangzhou Medical University) was used as a positive control, and BHI medium was saved as a negative control. The positive biofilm formation of an *S. haemolyticus* isolate was considered biofilm positive if it had an OD_570_ value ≥ 0.25. Three replicate wells were made for each experiment, and the experiment was repeated three times.

### Statistical analysis

All data were analyzed with GraphPad Prism 9.5 and presented as mean ± standard deviation (SD). Statistical analysis was performed by one-way analysis of variance (ANOVA). *P* < 0.05 was considered statistically significant.

## Data Availability

The sequencing data of 146 *Staphylococcus haemolyticus* isolates are available at NCBI BioProject under accession number PRJNA1155832.

## SUPPLEMENTAL MATERIAL

Supplemental figures spectrum.02577-24-s0001.docx Fig. S1 to S7.

Supplemental tables spectrum.02577-24-s0002.xlsx Tables S1 to S4.

ASM does not own the copyrights to Supplemental Material that may be linked to, or accessed through, an article. The authors have granted ASM a non-exclusive, world-wide license to publish the Supplemental Material files. Please contact the corresponding author directly for reuse.
