# Supplementary material for: Genomic and phenotypic characterization of multidrug-resistant Staphylococcus haemolyticus isolated from burn patients in Chongqing, southwestern China
Source: Microbiol Spectr. 2025 Apr 17;13(6):e02577-24. doi: 10.1128/spectrum.02577-24 (PMC12131843; doi:10.1128/spectrum.02577-24)
Supplement: Supplemental figures — Fig. S1 to S7. [file spectrum.02577-24-s0001.docx]

**Supplementary Figures**


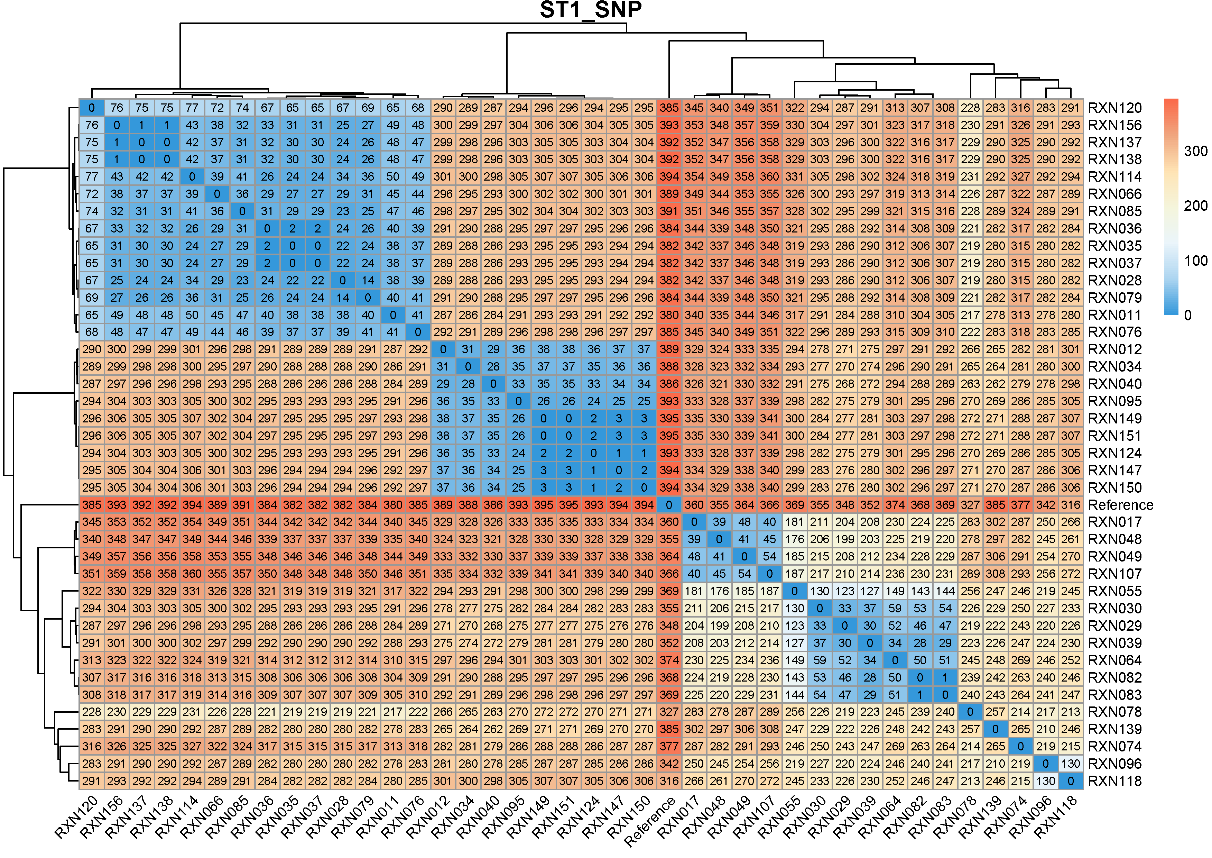


**Fig S1** SNP analysis of ST1 *S. haemolyticus* strains (*n* = 39). The reference strain was *S. haemolyticus* VB5326 (GenBank accession no.: GCA_009189185.2). Red to blue color scale in the heatmap reflects SNP density gradients (red: high; blue: low).


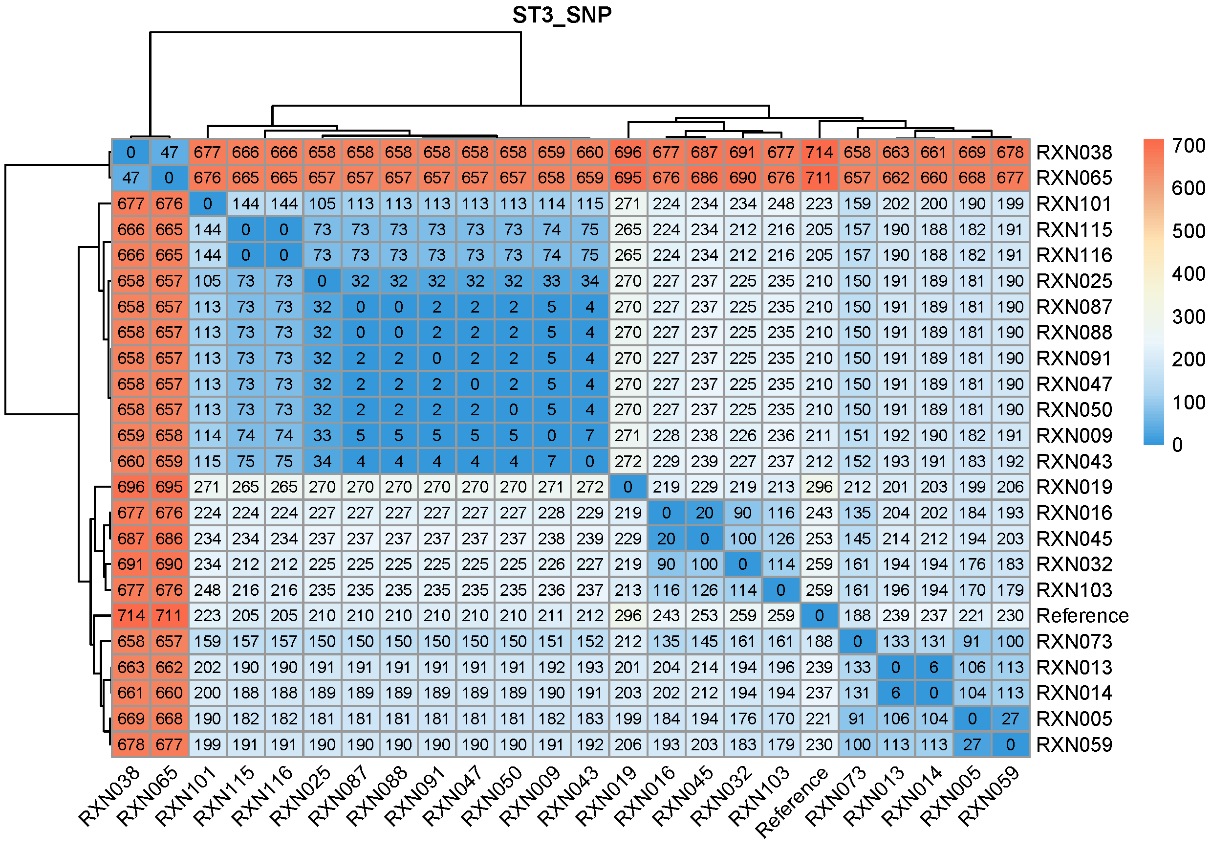


**Fig S2** SNP analysis of *S. haemolyticus* ST3 strains (n = 23). The reference strain was *S. haemolyticus* VB19458 (GenBank accession no.: GCA_003596365.3). Red to blue color scale in the heatmap reflects SNP density gradients (red: high; blue: low).


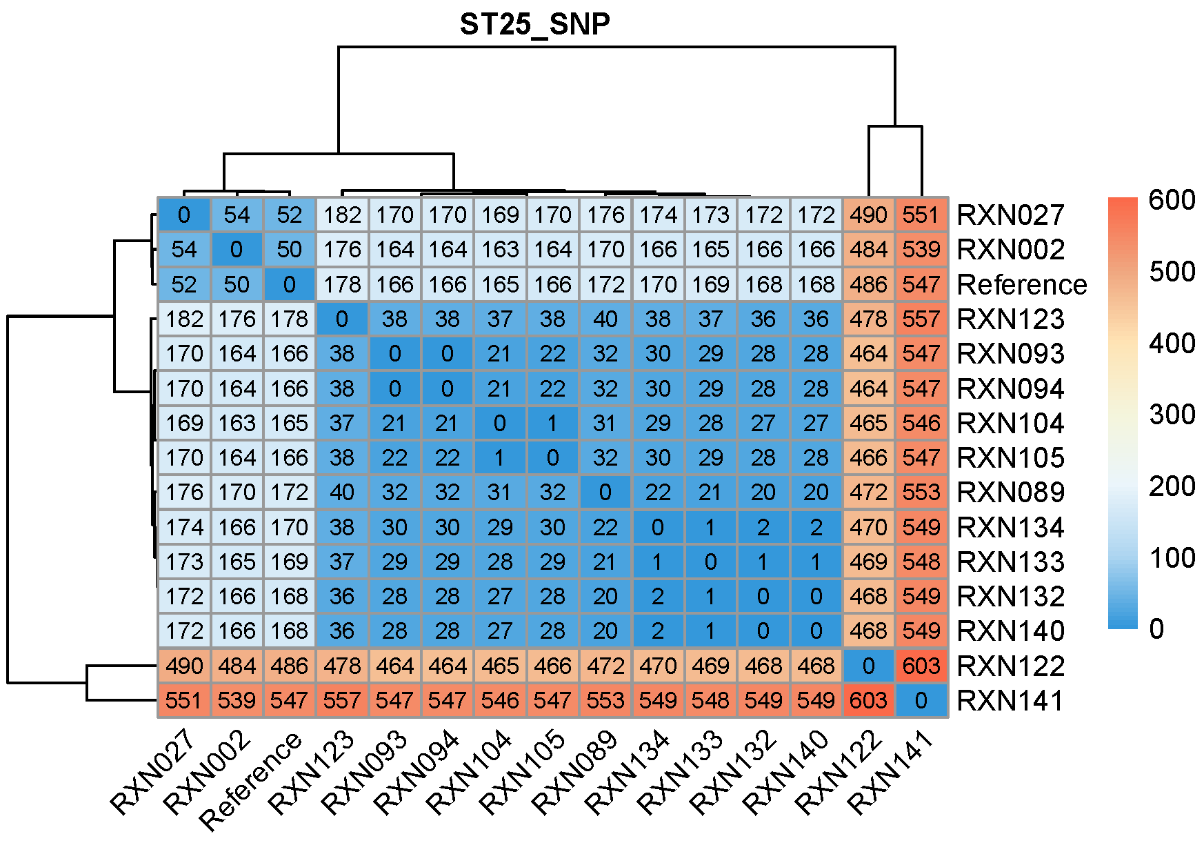


**Fig S3** SNP analysis of *S. haemolyticus* ST25 strains (n=14). The reference strain was *S. haemolyticus* 83131A (GenBank accession no.: GCA_002906595.1). Red to blue color scale in the heatmap reflects SNP density gradients (red: high; blue: low).


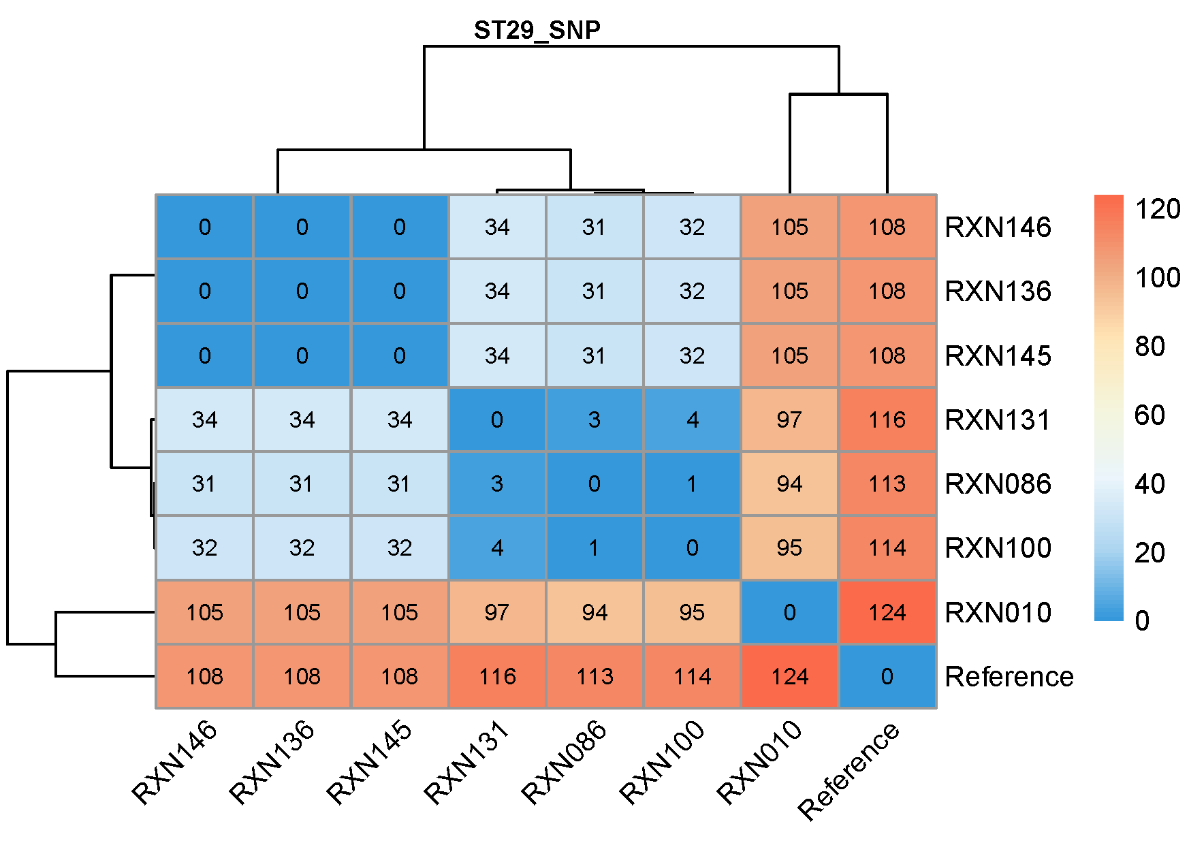


**Fig S4** SNP analysis of *S. haemolyticus* ST29 strains (*n* = 14). The reference strain was *S. haemolyticus* 7067_4#27 (GenBank accession no.: GCA_001234445.1). Red to blue color scale in the heatmap reflects SNP density gradients (red: high; blue: low).


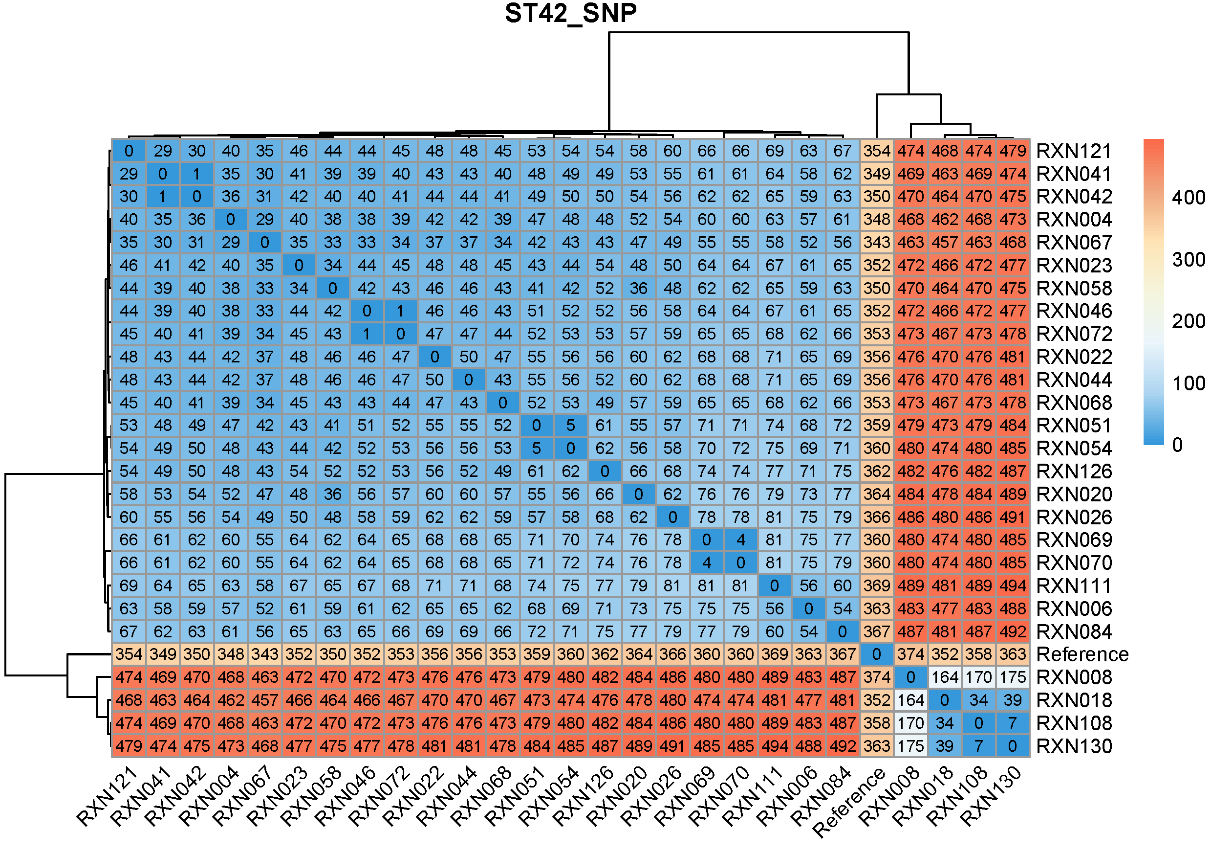


**Fig S5** SNP analysis of *S. haemolyticus* ST42 strains (*n* = 26). The reference strain was *S. haemolyticus* CGMH-SH51 (GenBank accession no.: GCA_027594625.1). Red to blue color scale in the heatmap reflects SNP density gradients (red: high; blue: low).


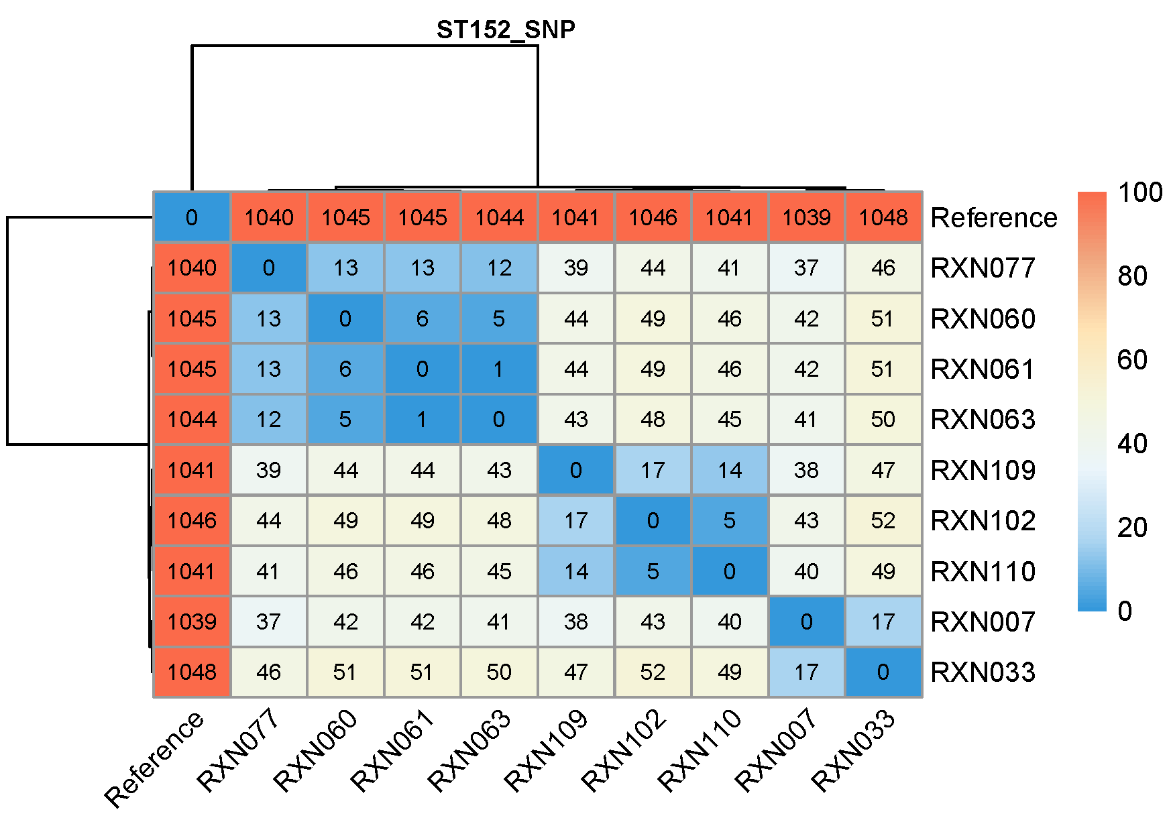


**Fig S6** SNP analysis of *S. haemolyticus* ST152 strains (*n* = 9). The reference strain was *S. haemolyticus* JCSC1435 (GenBank accession no.: GCA_000009865.1). Red to blue color scale in the heatmap reflects SNP density variations (red: high; blue: low).


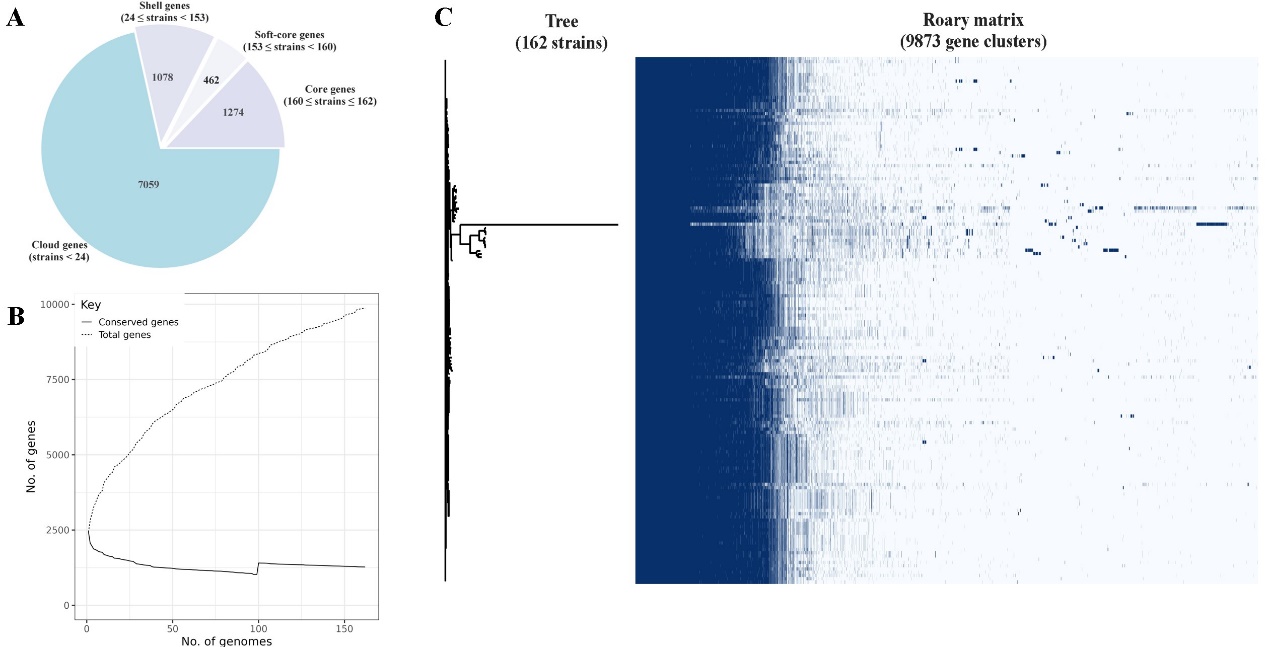


**Fig S7** Pan-genome analysis of *S. haemolyticus* isolates (*n* = 162). The strain information is provided in Table S3. (A) The size and distribution of the pan-genome into the subgroups; Core genes (99% ≤ strains ≤ 100%), Soft core genes (95% ≤ strains < 99%), Shell genes (15% ≤ strains < 95%), and Cloud genes (0% ≤ strains < 15%). (B) Gene accumulation curve analysis of *S. haemolyticus* isolates. The number of core genes is indicated with black full line, and the number of open pan-genomic genes is pointed by dashed line. (C) Gene distribution patterns of *S. haemolyticus* strains. Presence (blue) and absence (gray) were displayed. Genome ordering followed an ML tree constructed based on core genomic SNPs.
